# Serum Galectin-3 as a Non-Invasive Marker for Primary Sclerosing Cholangitis

**DOI:** 10.3390/ijms25094765

**Published:** 2024-04-27

**Authors:** Ganimete Bajraktari, Tanja Elger, Muriel Huss, Johanna Loibl, Andreas Albert, Arne Kandulski, Martina Müller, Hauke Christian Tews, Christa Buechler

**Affiliations:** Department of Internal Medicine I, Gastroenterology, Hepatology, Endocrinology, Rheumatology, and Infectious Diseases, University Hospital Regensburg, 93053 Regensburg, Germany; ganimete1.bajraktari@stud.uni-regensburg.de (G.B.); tanja.elger@klinik.uni-regensburg.de (T.E.); muriel.huss@klinik.uni-regensburg.de (M.H.); johanna.loibl@klinik.uni-regensburg.de (J.L.); andreas.albert@klinik.uni-regensburg.de (A.A.); arne.kandulski@klinik.uni-regensburg.de (A.K.); martina.mueller-schilling@klinik.uni-regensburg.de (M.M.); hauke.tews@klinik.uni-regensburg.de (H.C.T.)

**Keywords:** galectin-3, aminotransferase, calprotectin, urine, primary sclerosing cholangitis, inflammatory bowel disease

## Abstract

Primary sclerosing cholangitis (PSC) is a serious liver disease associated with inflammatory bowel disease (IBD). Galectin-3, an inflammatory and fibrotic molecule, has elevated circulating levels in patients with chronic liver disease and inflammatory bowel disease (IBD). This study aims to clarify whether galectin-3 can differentiate between patients with IBD, PSC, and PSC-IBD. Our study measured serum galectin-3 levels in 38 healthy controls, 55 patients with IBD, and 22 patients with PSC (11 patients had underlying IBD and 11 patients did not), alongside the urinary galectin-3 of these patients and 18 controls. Serum and urinary galectin-3 levels in IBD patients were comparable to those in controls. Among IBD patients, those with high fecal calprotectin, indicating severe disease, exhibited lower serum and elevated urinary galectin-3 levels compared to those with low calprotectin levels. Serum galectin-3 levels were inversely correlated with C-reactive protein levels. PSC patients displayed higher serum and urinary galectin-3 levels than IBD patients, with the highest serum levels observed in PSC patients with coexisting IBD. There was no correlation between serum and urinary galectin-3 levels and laboratory indicators of liver injury in both IBD and PSC patients. In conclusion, this study demonstrates that serum and urinary galectin-3 levels can distinguish IBD from PSC patients, and also reveals higher serum galectin-3 levels in PSC-IBD patients compared to those with isolated PSC.

## 1. Introduction

Primary sclerosing cholangitis (PSC) is a rare chronic biliary disease with a prevalence of approximately 6 per 100,000 person-years in the UK population [1]. This disease is progressive and is characterized by the destruction of the bile ducts, leading to cholestasis, liver fibrosis, and, ultimately, liver cirrhosis. Currently, there are no effective drugs identified to halt the progression of the disease. Diagnosing PSC can be challenging, as noted by Pria et al. [2]. The gold standard for initial non-invasive diagnostics is magnetic resonance cholangiopancreatography (MRCP) [3,4]. This cost-intensive imaging method also has uncertainties in the assessment of the bile ducts, which is why non-invasive markers are needed. Furthermore, it is advised that patients with IBD undergo annual liver function screening, irrespective of symptoms [5].

PSC is frequently linked to inflammatory bowel disease (IBD), with about 70% of PSC patients also suffering from IBD [5].The two main entities of IBD are Crohn’s disease (CD) and ulcerative colitis (UC) [6,7,8]. Despite extensive research identifying genetic factors, microbial dysbiosis, immune dysfunction, and environmental influences as contributors to IBD pathogenesis, its exact cause remains unclear [9]. 

Non-invasive diagnostic and prognostic biomarkers for PSC are currently an area of unmet need [10]. Biomarkers such as anti-neutrophil cytoplasmic antibodies (ANCAs) are suboptimal in their performance [10]. One study from Australia showed that more than 80% of IBD patients had a positive ANCA result and a significant proportion had proteinase 3 antibodies. However, no specific ANCA pattern predicted a specific IBD subtype or PSC [11]. Anti-glycoprotein 2 combined with ANCAs to serine proteinase 3 (PR3-ANCA) are prognostic in PSC for poor survival [12] but have still to be tested in larger cohorts.

Galectin-3 is expressed by various human cells, especially immune cells, adipocytes, epithelial cells, and endothelial cells, and serum galectin-3 levels are elevated in obesity [13,14,15]. Galectin-3 is an inflammatory and fibrotic molecule. Inflammation is known to be an important pathogenic event in liver injury, and reactive oxygen species (ROS) cause the induction of pro-inflammatory genes. Excessive production of ROS and overexpression of these pro-inflammatory proteins play an important role in the progression of liver disease severity [16,17]. Galectin-3 activates myofibroblasts, which produce excess extracellular matrix, resulting in scar formation. Mice deficient in galectin-3 were protected from carbon tetrachloride-induced liver fibrosis and non-alcoholic steatohepatitis (NASH) [18,19]. It has been suggested that galectin-3 plays a causal role in tissue fibrosis. In the liver, galectin-3 expression is induced in cirrhosis. Higher hepatic and serum galectin-3 levels have been reported in patients with chronic liver disease of various etiologies compared to liver-healthy controls [18,20,21,22,23,24,25]. In liver cirrhosis, hepatocyte galectin-3 levels are strongly induced, whereas in patients with cholestasis, mostly galectin-3 expression of Kupffer cells is upregulated. In the normal liver, galectin-3 is expressed in bile duct cells, and the galectin-3 protein levels of bile duct cells also seem to be increased in cholestasis and cirrhosis [26,27]. 

This has led to the development of galectin-3-blocking agents as anti-fibrotic drugs. Currently, the effects of galectin-3 inhibitors are being investigated in patients with liver fibrosis. However, the drugs tested so far have not been able to improve the fibrosis stage in patients with NASH [28].

Dextran sodium sulfate (DSS) is frequently used to induce IBD in mice by adding it to their drinking water. DSS disrupts the epithelial barrier, allowing intestinal bacteria to invade the mucosa, leading to immune cell infiltration and inflammation. A leaky gut leads to liver and bile duct inflammation, which can progress to cirrhosis. Impaired liver function and intestinal inflammation contribute to intestinal dysbiosis, which can further exacerbate the disease [29,30] (Figure S1). In the DSS model, a comparison between wild-type and galectin-3-deficient mice revealed that galectin-3 plays a role in resolving inflammation. Furthermore, acute DSS-induced colitis was ameliorated by galectin-3 treatment [31]. In both acute and chronic models of colitis, a peritoneal injection of recombinant galectin-3 significantly reduced colonic IL-6 levels [32]. 

It is worth noting that serum galectin-3 levels in patients with UC showed a negative correlation with endoscopic and histological parameters of colitis. Additionally, elevated fecal galectin-3 levels were found to be an indicator of UC remission [31].

Recent studies observed increased serum galectin-3 levels in IBD, which were unable to distinguish between active disease and remission in both UC and CD patients [33,34]. This suggests that galectin-3 may not be a reliable biomarker for disease activity in IBD. It has also been reported that serum galectin-3 levels did not differ between IBD patients and controls [35]. To summarize, current evidence is inconclusive about systemic levels of galectin-3 and its association with disease activity in IBD. 

Urinary proteins are increasingly recognized as biomarkers for a range of diseases [36]. Elevation of galectin-3 in urine may serve as a marker for progressive kidney injury and renal fibrosis. Additionally, it has been identified as a biomarker for heart failure and various cancers [36,37,38].

Renal manifestations are present in approximately 6% of patients with IBD. The most frequent type of renal involvement is nephrolithiasis [39,40]. IBD patients with renal involvement had a lower glomerular filtration rate and higher serum creatinine levels compared to IBD patients with normal renal function [40]. Nephrolithiasis is twice as common in patients with chronic liver disease in comparison to healthy individuals [41].

Galectin-3 in serum can indicate liver diseases. However, it has not been evaluated whether it can distinguish between PSC and IBD. Therefore, in this study, the serum galectin-3 levels of healthy controls, patients with IBD, and patients with PSC were measured to address this issue. In addition, the potential of urinary galectin-3 as a biomarker for PSC was analyzed.

## 2. Results

### 2.1. Serum and Urinary Galectin-3 of IBD Patients

The cohort included 55 IBD patients, 22 PSC patients (11 patients had underlying IBD and 11 patients did not), and 38 controls. The controls and patients had a comparable age and gender distribution (Table 1). The PSC patients had higher serum creatinine and a lower glomerular filtration rate (GFR) than the IBD patients. Aspartate aminotransferase (AST), gamma glutamyl transferase (gamma GT), alkaline phosphatase (AP) and bilirubin were elevated in PSC compared to IBD patients (Table 1). The urinary protein/creatinine levels of controls and patients with IBD or PSC were similar. The cutoff value for proteinuria is 0.2 [42], showing that our controls and patients had normal renal function.

In the control cohort, serum galectin-3 was negatively correlated with age (r = −0.363, *p* = 0.025). No such association was observed for urinary galectin-3 (r = 0.439, *p* = 0.101). Serum galectin-3 was higher in women than in men (*p* = 0.054), whereas urinary galectin-3 was similar in both sexes (*p* = 0.814).

In IBD patients, serum and urine galectin-3 did not correlate with age (r = −0.080, *p* = 0.562 and r = 0.008, *p* = 0.954, respectively) and BMI (r = −0.054, *p* = 0.705 and r = 0.057, *p* = 0.708, respectively). Males and females had similar serum (*p* = 0.986) and urinary (*p* = 0.821) galectin-3 levels. 

**Figure 1:**
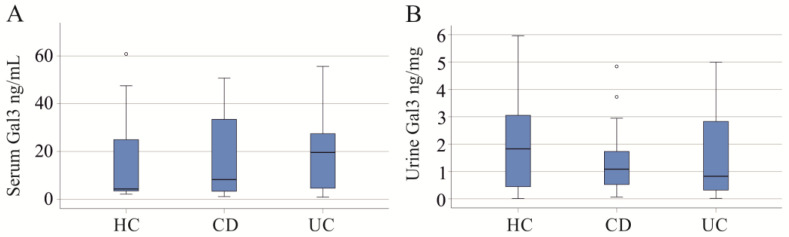
Serum and urinary galectin-3 (Gal3) of healthy controls (HC) and patients with Crohn’s disease (CD) and ulcerative colitis (UC). (**A**) Serum Gal3; (**B**) urinary Gal3. Small circles in the figures are outliers.

Serum and urine galectin-3 in IBD were not correlated (r = −0.083, *p* = 0.566). The IBD cohort included 36 patients with CD and 19 patients with UC. Serum and urinary galectin-3 were similar between these two groups (Figure 1A,B).

**Figure 2:**
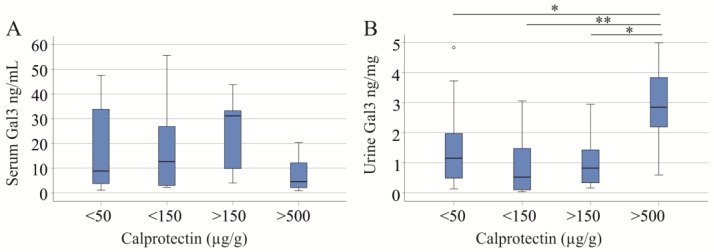
Serum and urinary galectin-3 (Gal3) in relation to fecal calprotectin: (**A**) serum Gal3; (**B**) urinary Gal3. * *p* < 0.05, ** *p* < 0.01. The small circle in the (**B**) is an outlier.

Stratifying IBD patients by fecal calprotectin levels showed no significant difference in serum galectin-3 levels across groups (*p* = 0.168; Figure 2A). Specifically, 27 patients had fecal calprotectin levels < 50 µg/g, 11 had levels between 50 and 150 µg/g, 8 had levels between 150 and 500 µg/g, and 7 had levels > 500 µg/g, with data for 2 patients undocumented. Nonetheless, a trend toward lower serum galectin-3 levels was observed in patients with high fecal calprotectin compared to those with low levels (*p* = 0.056). Of note, urinary galectin-3 levels were significantly different (*p* = 0.024) and increased in patients with high calprotectin (Figure 2B). Serum creatinine (*p* = 0.639) and GFR (*p* = 0.073) were not changed with increasing levels of fecal calprotectin. 

In IBD, galectin-3 in serum and urine did not correlate with creatinine, GFR, and fecal calprotectin. Serum galectin-3 was negatively correlated with CRP (Table 2). ALT, AST, gamma GT, and AP correlated with neither the serum nor urinary galectin-3 of IBD patients (*p* > 0.05 for all). 

Galectin-3 has been described as a diagnostic biomarker for tumors [43]. In our IBD patients, 10 patients (of the 52 patients where this was documented) developed tumors during therapy. Serum galectin-3 (*p* = 0.296) was similar between IBD patients with and without tumors. The urinary galectin-3 of patients with tumors tended to be increased (*p* = 0.141) (Figure S2). 

Intestinal fistulas are mostly a complication of CD [44], but IBD patients with fistulas (14 patients) had similar serum (*p* = 0.323) and urine (*p* = 0.441) galectin-3 levels to patients without fistulas. 

### 2.2. Serum and Urinary Galectin-3 of PSC Patients

**Figure 3:**
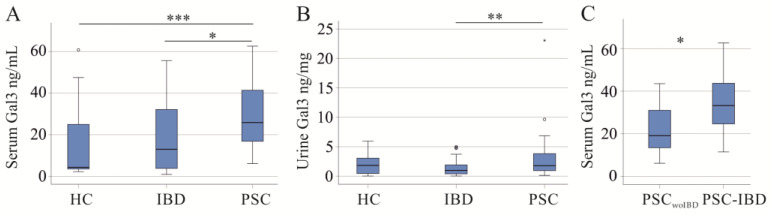
Serum and urinary galectin-3 (Gal3) of healthy controls (HC), patients with inflammatory bowel disease (IBD) and patients with primary sclerosing cholangitis (PSC). (**A**) Serum Gal3; (**B**) urinary Gal3; (**C**) serum Gal3 of PSC patients without IBD (PSC_woIBD_) and PSC patients with IBD (PSC-IBD). * *p* < 0.05, ** *p* < 0.01, *** *p* < 0.001. Small circles and asterisk in the figure are outliers.

PSC is a liver disease associated with IBD [5], and serum galectin-3 was higher in PSC compared to IBD patients and healthy controls (Figure 3A). The area under the receiver operating characteristic (AUROC) for discrimination of PSC and IBD was 0.673. 

Urinary galectin-3 of PSC patients was induced in comparison to IBD patients but was similar to healthy controls (Figure 3B). The AUROC for discrimination of PSC and IBD patients was 0.696. 

The 11 PSC-IBD patients had higher serum galectin-3 than the 11 PSC patients without underlying IBD (PSC_woIBD_) (*p* = 0.033) (Figure 3C). Urinary galectin-3 was comparable (*p* = 0.751).

**Figure 4:**
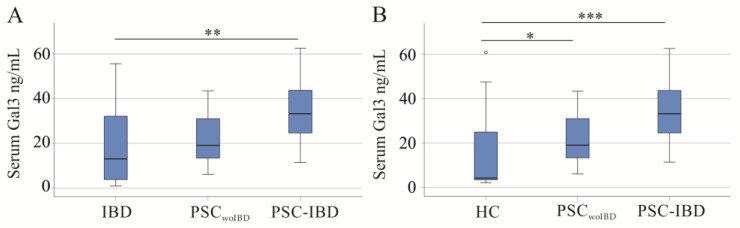
Serum galectin-3 (Gal3) of healthy controls (HC), patients with inflammatory bowel disease (IBD), patients with primary sclerosing cholangitis without underlying IBD (PSC_woIBD_), and PSC-IBD patients: (**A**) Serum Gal3 of IBD, PSC_woIBD_, and PSC-IBD patients; (**B**) serum Gal3 of HC, PSC_woIBD_, and PSC-IBD patients. * *p* < 0.05, ** *p* < 0.01, *** *p* < 0.001. Small circle in the figure is an outlier.

Galectin-3 serum levels of PSC_woIBD_ patients were similar to IBD patients, and were significantly higher in PSC-IBD (Figure 4A). The AUROC for discrimination of PSC-IBD from IBD was 0.760. PSC_woIBD_ and PSC-IBD patients had similar calprotectin, CRP, creatinine, and GFR (*p* > 0.05). 

Galectin-3 serum levels of PSC_woIBD_ and PSC-IBD were higher compared to healthy controls (Figure 4B). 

In PSC patients, galectin-3 in both serum and urine did not correlate with creatinine, GFR, CRP, and fecal calprotectin (Table 3). ALT, AST, gamma GT, AP, and the MELD score correlated with neither the serum nor urinary galectin-3 of PSC patients (*p* > 0.05 for all). 

In PSC-IBD patients, a negative correlation of serum galectin-3 and CRP (r = −0.652, *p* = 0.041) was observed. All other laboratory measures did not significantly correlate with the serum or urinary galectin-3 of PSC-IBD and PSC_woIBD_ patients (*p* > 0.05 for all). 

## 3. Discussion

In our study, we analyzed galectin-3 levels in serum and urine, finding that serum galectin-3 levels can distinguish between IBD and PSC-IBD patients. Notably, PSC-IBD patients exhibit higher serum galectin-3 levels compared to both PSC without IBD (PSC_woIBD_) and IBD patients alone, suggesting a pathophysiological and clinical relevance.

Experimental studies in mice with colitis have mostly provided evidence for a protective role of galectin-3 [31,32,45]. Current evidence about systemic levels of galectin-3 in IBD and its association with disease activity is, however, inconclusive [31,33,34,35]. In our cohort, serum and urinary galectin-3 levels were similar in IBD patients and controls, and did not differ between UC and CD patients. IBD patients with high fecal calprotectin levels showed a decrease in serum galectin-3 and a concomitant increase in urinary galectin-3. Creatinine and GFR did not change in patients with massive inflammation, and renal disease did not appear to cause this change. The negative correlation of serum galectin-3 with CRP in IBD is consistent with a decrease in serum galectin-3 levels in active disease. Reduced serum galectin-3 in patients with higher disease activity has been reported by Volarevic et al. [31], but, e.g., Yu et al. did not identify different galectin-3 serum levels of IBD patients with active and inactive disease [34]. However, it is difficult to compare the disease severity of patients enrolled in different studies. Thus, some of our IBD patients may have more severe disease and correspondingly lower serum galectin-3 levels compared to patients enrolled in other studies. The median fecal calprotectin level of our patients was 50 µg/g and was higher than that of the patients in the study by Volarevic et al. [31], where most patients had calprotectin levels below 25 µg/g. It remains to be seen whether fecal calprotectin levels determined by different assays can be compared.

PSC is often associated with IBD [5,46]. The diagnosis of PSC remains challenging and there is currently no non-invasive biomarker available [46,47]. As serum galectin-3 is elevated in patients with chronic liver disease [20], it can be hypothesized that serum galectin-3 may discriminate between IBD and PSC. Serum and urinary galectin-3 levels were indeed higher in PSC compared to IBD patients. Serum galectin-3 was also elevated in PSC patients compared to healthy controls, in agreement with previous studies [20,21,25].

It is of particular interest that serum and urinary galectin-3 levels are not associated with elevated ALT and AST levels, yet are clearly associated with PSC. 

PSC patients with underlying IBD had higher serum galectin-3 than patients with isolated PSC. Previous studies suggested a protective role of galectin-3 in IBD [32,48], and higher levels in PSC-IBD may thus contribute to less severe IBD, commonly described in patients with PSC-IBD [5]. Whether serum galectin-3 can be used as a biomarker to differentiate patients with isolated PSC from patients with PSC-IBD is a matter for future research. PSC_woIBD_ patients have a more rapid progression of liver fibrosis compared to PSC-IBD patients, who have a higher risk of malignancy [49]. 

Galectin-3 plays a role in several diseases and has been described as a biomarker for different types of cancer, liver disease, kidney disease, and heart failure [36,43]. In IBD, serum galectin-3 was not increased in patients who developed tumors during therapy. Urinary galectin-3 was slightly higher, but this effect was not significant. The morbidity pattern among middle-aged and older individuals in Germany was evaluated in 2023. This study identified clinically meaningful multimorbidity classes (their prevalence is given in brackets) such as the arthrosis/inflammation/mental illness class (21%), the hypertension/metabolic class (22%), and the cardiovascular/cancer class (2%) [50]. Although not documented for our cohorts, those patients likely had a similar or even higher range of comorbidities [51]. However, further studies are needed to evaluate a possible confounding effect of the above-mentioned comorbidities on serum and urinary galectin-3 in IBD and PSC. 

In females, higher serum galectin-3 levels were observed compared to men [52], and this was also evident in our control cohort. A sex-specific difference of serum galectin-3 level was not detected in our patients. The study by Boer et al. including approximately 8000 subjects from the general population described a positive correlation of plasma galectin-3 with age [52], whereas this association was negative in our controls. In our patient cohort, serum galectin-3 levels were not found to be related to age. Although higher galectin-3 levels were present in overweight/obese subjects, galectin-3 did not correlate with BMI in IBD patients [15,52]. Age, sex, and BMI are, therefore, not regarded as confounding factors for the analysis of galectin-3 in the serum and urine of IBD and PSC patients. 

A previous study suggested that higher plasma galectin-3 levels are due to impaired renal excretion of galectin-3 [53]. In the IBD cohort, the high serum galectin-3 of patients with active disease was indeed related with low galectin-3 levels in urine. Otherwise, we could not detect correlations between serum and urinary galectin-3 levels. In PSC patients, both serum and urinary galectin-3 levels were elevated. The urinary protein/creatinine ratio is a measure of proteinuria [42] and was normal in our patients. This shows that impaired renal galectin-3 excretion is not the main cause of higher serum galectin-3 levels. 

This study has limitations. The samples were collected at only one time point, and the number of patients with PSC_woIBD_ and PSC-IBD, which are rare diseases, was small. Additionally, comorbidities such as diabetes or cardiovascular diseases were not documented. Urine and blood samples have been collected at any time during the day, which is an advantage for clinical tests. The significance of our study is to suggest studies evaluating continuous monitoring of galectin-3 for the diagnosis of PSC. A potential pathogenic role of galectin-3 in PSC should also be evaluated. 

## 4. Materials and Methods

### 4.1. Patients and Control Cohorts

From 6 December 2021 to 31 January 2023, patients diagnosed with IBD or PSC (11 patients had underlying IBD and 11 patients did not) at the Department of Internal Medicine I (University Hospital of Regensburg) were recruited. Diagnosis of IBD and PSC was based on histologic, endoscopic, and clinical criteria [54,55,56]. In the IBD cohort, 12 patients were treated with corticosteroids, 19 with mesalazine, 17 with anti-interleukin 12/23 antibodies, 18 with anti-TNF antibodies, and 7 with azathioprine. The 22 PSC patients in our study were treated with ursodeoxycholic acid. Patients with coagulopathy were excluded from the study. The serum and urine of patients, as well as controls, were collected and stored at −80 °C until use. For this retrospective study, the controls were students, hospital staff, and partners of the patients who lived in the same area as the patients with IBD. 

### 4.2. Measurement of Galectin-3, Urinary Creatinine, and Urinary Protein

The ELISA to measure urinary galectin-3 was from AOBIOUS INC. (Cat. No.: E0497h; Gloucester, MA, USA). Urine was used undiluted. For analysis of serum galectin-3, serum was diluted 1:3-fold in Reagent Diluent as recommended by the provider of the ELISA (Cat. No.: DY1154; R&D Systems, Wiesbaden-Nordenstadt, Germany). Urinary creatinine was measured by the creatinine parameter assay kit (Cat. No.: KGE005; R&D Systems) in 1:20-fold diluted urine. Urinary galectin-3 levels relative to urinary creatinine levels were used for calculations. Pierce™ BCA Protein Assay Kits (Cat. No.: 23225, Thermo Fisher Scientific, Waltham, MA, USA) were used to determine urinary protein concentrations using 1:10-diluted spot urine samples of our patients and controls. 

### 4.3. Collection of Urine

Urine was collected during the day, aliquoted into appropriate portions, and stored at −80 °C.

### 4.4. Statistical Analysis

Data are shown as boxplots and outliers are marked as circles and asterisks. A receiver operating characteristic curve, Mann–Whitney U-test, Kruskal–Wallis Test, and Spearman correlation were the statistical tests used (SPSS Statistics 26.0 program, IBM, Leibniz Rechenzentrum, München, Germany). A value of *p* < 0.05 was regarded as significant. 

## 5. Conclusions

This study demonstrates that serum galectin-3 levels differentiate between IBD, PSC, and PSC-IBD patients. Notably, individuals with both PSC and IBD exhibit elevated serum galectin-3 levels compared to those with PSC alone, suggesting potential pathophysiological implications. Thus, serum galectin-3 emerges as a promising novel biomarker for PSC. PSC is difficult to diagnose, and no specific biomarker that could support the diagnosis of PSC has been identified [10,57]. Liver function tests may be useful to start an investigation, but there are no specific laboratory markers that help diagnose PSC [58]. Although patients with PSC may be initially assessed with ultrasound or computed tomography, cholangiography based on magnetic resonance cholangiography or endoscopic retrograde cholangiopancreatography evaluation is usually required to make a definitive diagnosis. Liver biopsy is only needed in suspected cases of small duct PSC when the cholangiogram is normal, or to confirm an overlap with autoimmune hepatitis [57]. Biomarkers are non-invasive and comparatively cheap analysts without the drawbacks of liver biopsy. However, a group of markers related to the different aspects of a disease are requested for clinical purposes. Galectin-3 may become one marker of a biomarker panel for the diagnosis of PSC. Moreover, increased galectin-3 in PSC may also initiate studies to evaluate galectin-3-blocking agents in cholestatic liver diseases. 

## Figures and Tables

**Table 1:** Characteristics of the study groups. The PSC cohort included 11 patients with PSC and IBD and 11 patients with PSC without underlying IBD (PSC_woIBD_). The IBD cohort does not include patients with PSC. Data are reported as median, minimum, and maximum values. The Model for End Stage Liver Disease (MELD) score was documented for PSC patients. Urine protein/creatinine ratio of 18 controls has been determined. The statistical test used: Kruskal–Wallis Test (alanine aminotransferase (ALT), alkaline phosphatase (AP), aspartate aminotransferase (AST), gamma glutamyl transferase (gamma GT), glomerular filtration rate (GFR), not determined (n.d.)). * *p* < 0.05, ** *p* < 0.01, *** *p* < 0.001.

Characteristics	IBD	PSC	Controls
Number (females/males)	55 (23/32)	22 (6/16)	38 (20/18)
Age (years)	47 (19–70)	52 (18–70)	55 (23–78)
BMI (kg/m^2^)	25.1 (15.5–40.4)	24.8 (18.0–31.8)	n.d.
C-reactive protein (mg/L)	3 (0–144)	2 (0–51)	n.d.
Creatinine (mg/dL)	0.83 (0.51–1.25) *	1.02 (0.68–3.94) *	n.d.
GFR (mL/min)	100 (61–136) *	86 (12–135) *	n.d.
Fecal calprotectin (µg/g)	49 (0–3889)	35 (0–999)	n.d.
AST (U/L)	25 (10–41) *	27 (15–177) *	n.d.
ALT (U/L)	20 (7–63)	27 (5–89)	n.d.
Gamma GT (U/L)	26 (11–74) *	54 (10–345) *	n.d.
AP (U/L)	64 (43–142) ***	117 (57–537) ***	n.d.
Bilirubin (mg/dL)	0.50 (0.15–1.90) **	0.70 (0.20–14.00) **	n.d.
MELD Score	n.d.	6 (6–20)	n.d.
Urine protein/creatinine	0.05 (0–14.89)	0.08 (0–4.60)	0.05 (0 –0.20)

**Table 2:** Spearman correlation of serum and urinary galectin-3 with creatinine, glomerular filtration rate, C-reactive protein, and fecal calprotectin in IBD.

Creatinine	Glomerular Filtration Rate	C-Reactive Protein	Fecal Calprotectin
Serum Galectin-3			
r = 0.049	r = 0.002	r = −0.284	r = −0.102
*p* = 0.727	*p* = 0.989	*p* = 0.043	*p* = 0.463
Urinary Galectin-3			
r = −0.203	r = 0.126	r = 0.107	r = 0.090
*p* = 0.161	*p* = 0.387	*p* = 0.474	*p* = 0.539

**Table 3:** Spearman correlation coefficients for the correlation of serum and urinary galectin-3 with creatinine, glomerular filtration rate, C-reactive protein, and fecal calprotectin in patients with PSC.

Creatinine	Glomerular Filtration Rate	C-Reactive Protein	Fecal Calprotectin
Serum Galectin-3			
r = 0.091	r = 0.035	r = −0.335	r = 0.105
*p* = 0.803	*p* = 0.914	*p* = 0.287	*p* = 0.759
Urinary Galectin-3			
r = −0.145	r = −0.049	r = −0.145	r = −0.138
*p* = 0.592	*p* = 0.858	*p* = 0.592	*p* = 0.623

## Data Availability

Original research data can be obtained on request.

## Supplementary Materials

The following supporting information can be downloaded at https://www.mdpi.com/article/10.3390/ijms25094765/s1.
