# Supplementary material for: Serum Galectin-3 as a Non-Invasive Marker for Primary Sclerosing Cholangitis
Source: Int J Mol Sci. 2024 Apr 27;25(9):4765. doi: 10.3390/ijms25094765 (PMC11084718; doi:10.3390/ijms25094765)
Supplement: Supplementary file 1 [file ijms-25-04765-s001.zip › ijms-2952664-supplementary.pdf]

Serum Galectin-3 as a Non-Invasive Marker for Primary Sclerosing Cholangitis

Ganimete Bajraktari, Tanja Elger, Muriel Huss, Johanna Loibl, Andreas Albert, Arne Kandulski, Martina Müller, Hauke Christian Tews and Christa Buechler *

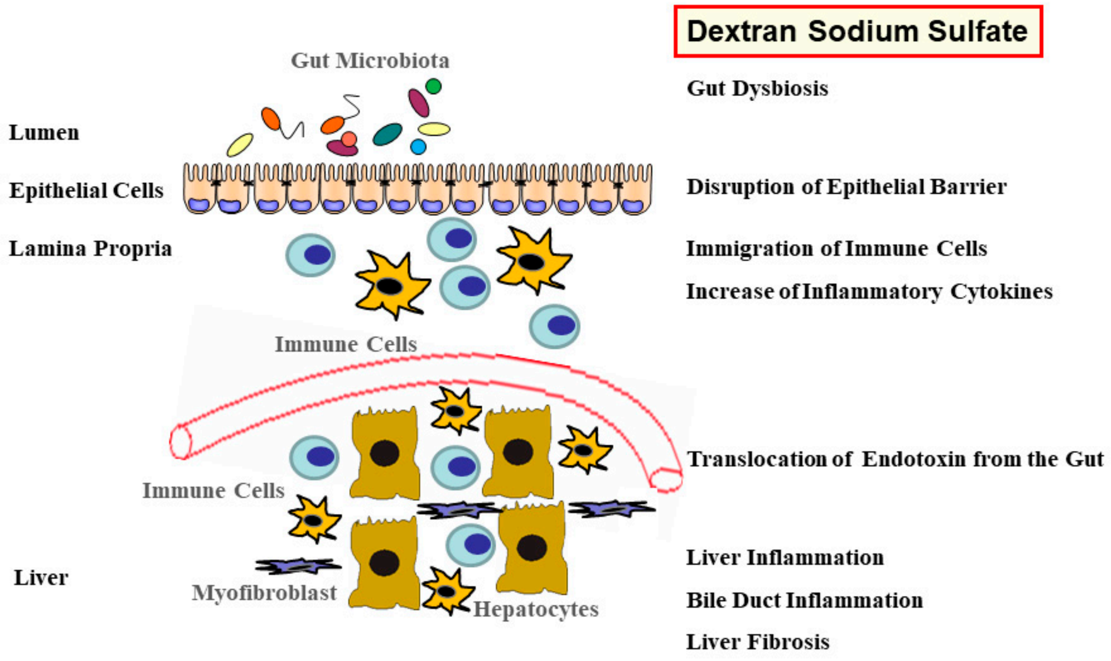

Figure S1. Schematic summary of the effect of dextran sodium sulfate in the gut and the liver.

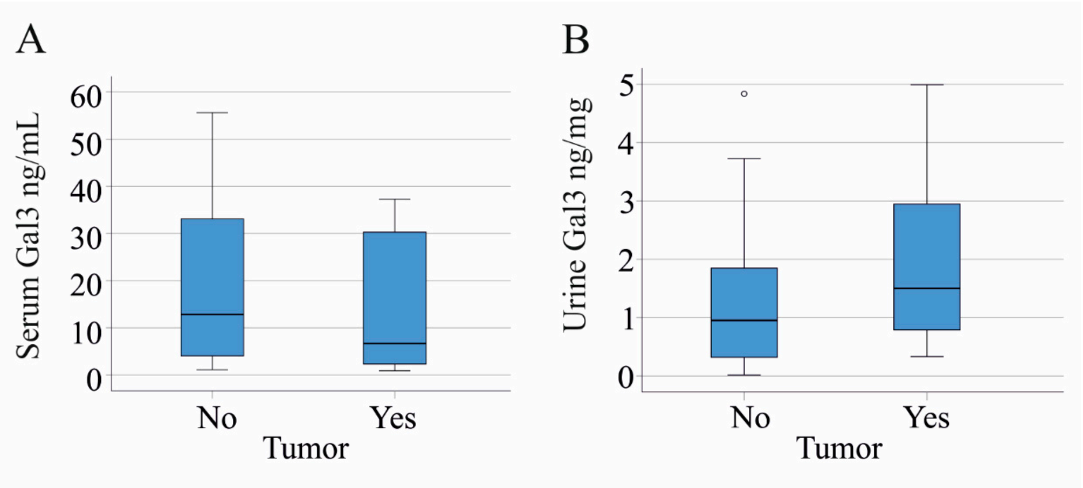

Figure S2. Serum and urinary galectin-3 (Gal3) of patients with inflammatory bowel disease who did develop tumors during therapy in comparison to patients who did not have tumors. (**a**) Serum Gal3; (**b**) Urinary Gal3.
